# Efficacy of 10% imidacloprid + 2.5% moxidectin topical solution (Advantage Multi^®^ for Dogs) for the prevention of heartworm disease and infection all month long

**DOI:** 10.1186/s13071-017-2432-x

**Published:** 2017-11-09

**Authors:** Dwight D. Bowman, Cameon M. Ohmes, Joseph A. Hostetler, Daniel J. Keil, Terry L. Settje, Samuel D. Charles

**Affiliations:** 1 Liberty Research Inc, Waverly, NY USA; 2 Bayer Animal Health, Shawnee, KS USA; 3 Department of Microbiology and Immunology, College of Veterinary Medicine, Cornell University, 930 Campus Road, Ithaca, NY 14853 USA

**Keywords:** Advantage Multi® for Dogs, *Dirofilaria immitis*, Heartworm disease, All-month protection, Forward protection, Persistent efficacy, 30-Day protection, Proactive prevention, Imidacloprid, moxidectin, Advocate®

## Abstract

**Background:**

Prior work has shown that the levels of moxidectin in dogs treated with Advantage Multi® for Dogs (Bayer Animal Health) remain at a high plasma concentration for the full month after application. The objective of this study was to demonstrate the efficacy of 10% imidacloprid + 2.5% moxidectin topical solution (Advantage Multi® for Dogs, also known as Advocate® for Dogs) for the prevention of heartworm infection and disease 30 days after just one application.

**Methods:**

Two groups of eight dogs each were included. Dogs in Group 1 received the product (Advantage Multi® for Dogs) while those in Group 2 remained as nontreated controls. All dogs entering the study completed a physical examination including examination for *Dirofilaria immitis* antigen and circulating microfilariae. Dogs in Group 1 were treated on Study Day (SD) –30 as per the label recommendation. Thirty days later (SD 0) dogs in Groups 1 and 2 were subcutaneously infected in the inguinal region with approximately 50 infective third-stage *D. immitis* larvae (“Missouri” isolate). Blood was collected on SDs 120 and 147 for examination for *D. immitis* antigen and circulating microfilariae. On SD 148, all animals were euthanized and necropsied for recovery of adult heartworms. All procedures were performed in accordance with the VICH GL9 guidelines.

**Results:**

Examination and worm counts made at necropsy showed no heartworms in the treated dogs (Group 1) compared with six of eight nontreated dogs (Group 2) with heartworms (range of 2–33). The treated dogs (Group 1) had significantly fewer heartworms (*p* < 0.05) compared with the nontreated controls (Group 2).

**Conclusion:**

The results demonstrated that 10% imidacloprid + 2.5% moxidectin topical solution (Advantage Multi® for Dogs) is efficacious for the prevention of heartworm infection and disease all month long with no observation of treatment-related adverse events.

## Background

Persistent efficacy of topical imidacloprid + moxidectin (Advantage Multi®, Bayer Animal Health) was first demonstrated in dogs and cats against hookworm infections. In 2003, it was shown that when eight dogs were administered 10% imidacloprid + 2.5% moxidectin topical solution and subsequently infected with 300 *Uncinaria stenocephala* 18 days later, neither immature nor mature adult hookworms were present 21 days after infection, while the eight nontreated dogs harbored a mean of 4.6 (SD ± 3.9) immature adults and 8.1 (SD ± 4.3) mature adults [1]. In 2008, a similar study with hookworms of the genus *Ancylostoma* was reported in which cats and dogs were respectively administered each month, as per label, either five monthly treatments of 10% imidacloprid + 1% moxidectin topical solution (Advantage Multi® for Cats) or four monthly treatments of 10% imidacloprid + 2.5% moxidectin topical solution (Advantage Multi® for Dogs) [2]. Twenty (20) days after the last treatment, the six treated and six nontreated cats were orally infected with *Ancylostoma tubaeforme* larvae. Necropsy examinations conducted after establishment of patent infections in the nontreated cats showed none of the six treated cats had hookworms, while a mean of 62.5 adult *A. tubaeforme* was recovered from the nontreated cats. Each of the six treated and six nontreated dogs were orally infected with *U. stenocephala* larvae and *Ancylostoma caninum* larvae 22 and 23 days, respectively, after the fourth treatment. Necropsy examinations conducted after establishment of patent infections in the non-treated dogs showed all six treated dogs had no adult *U. stenocephala*, while a mean of 517.8 adult *U. stenocephala* was recovered from the nontreated control dogs. One adult *A. caninum* was recovered from one treated dog, while a mean of 41.3 *A. caninum* was recovered from the nontreated dogs.

Two studies evaluated the persistent efficacy of the pretreatment of cats and dogs with topical imidacloprid + moxidectin against heartworm infection. A study with 19 cats examined the effects of four monthly treatments (at 28-day intervals) followed by infection of each cat with 25 infective-stage *Dirofilaria immitis* larvae at 7, 14, 21, and 28 days after the last treatment [3]. Seven and one-half months after the fourth monthly treatment there were no heartworms recovered at necropsy from any of the 10 treated cats while a mean of 1.33 heartworms (range 1–6) was recovered from the nine nontreated cats. A study with a similar design was performed in 16 dogs (eight treated and eight nontreated dogs) in which the treated dogs had received four monthly treatments (at 28-day intervals) and then were infected with 50 third-stage *D. immitis* larvae 28 days after the fourth monthly treatment [4]. Again, there were no heartworms recovered from any of the treated dogs at necropsy conducted 5 months post infection, while a mean of 33.9 (range 25–41) heartworms was recovered from the eight nontreated dogs. These two studies demonstrated that four monthly applications of imidacloprid + moxidectin topical solution afforded protection to dogs and cats against subsequent heartworm infection for 4 weeks.

Previous publications evaluating the pharmacokinetic profile of moxidectin demonstrated that the repeated monthly application of topical imidacloprid + moxidectin to dogs and cats produces a continuous high level (steady-state) of protective moxidectin, month to month, after four or five repeated doses and also appears to provide protection for at least 28 days after the last administration against hookworms and heartworms in dogs and cats [2–4]. In the above referenced dog study, the authors postulated that due to this unique pharmacokinetic profile and high serum concentrations protection for 30 days against heartworms after just one dose is probable. The objective of this study was to determine whether a single application of 10% imidacloprid + 2.5% moxidectin topical solution (Advantage Multi® for Dogs) would protect dogs against an incoming *D. immitis* larval challenge 30 days after a single topical application of the product.

## Methods

### Ethical approval, animals, and animal care

The study was approved by the facility Institutional Animal Care and Use Committee (IACUC) with the concurrence of Bayer Animal Health and followed the guidelines of VICH GL9 (2001) [5]. The study included 16 purpose-bred mongrel dogs (six males and ten females) that were born and raised at the study facility that were approximately 7 months old at the beginning of the study. The dogs were maintained in indoor runs during the study. Nontreated dogs were housed in a separate room so that there was no contact with the treated dogs. Temperature was controlled by forced fan heat or air conditioning as needed. The dogs were fed a daily ration of Laboratory Canine Diet 5006 (LabDiet) in quantities sufficient for growth and maintenance. Dogs were provided water, originating from the municipal water supply, via individual automated watering system in each cage. All dogs were observed once daily as part of a general health observation with the exception of the day of treatment, Study Day (SD) –30, when the dogs were observed at 0.5, 1, 2, 4, and 8 h (+/−15 min) post treatment. Animals were weighed on SD −31 for ranking, randomization, and dose determination for SD −30.

Blood samples were collected from the study animals on SD –37, 120, and 147 for *D. immitis* antigen and microfilariae testing. Antigen testing was performed using the DiroCHEK® heartworm antigen test (Zoetis). Microfilariae testing was performed using the modified Knott test.

### Treatment

Using the body weights collected on SD −31, the 16 dogs were randomized to two treatment groups. Eight dogs in Group 1 were treated with 10% imidacloprid + 2.5% moxidectin topical solution as per label instructions and dosage recommendations on SD −30, while eight dogs in Group 2 remained untreated (Table 1).

**Table 1:** Treatment information on study animals in the 10% imidacloprid + 2.5% moxidectin (Advantage Multi®) and nontreated groups

Treatment group	Gender	Dog ID#	Study day	Body weight (pounds)	Dose (mL)
Group 1: 10% imidacloprid + 2.5% moxidectin	Female	1531603	−30	29.0	2.5
	Female	1531805	−30	16.6	1.0
	Female	1531806	−30	18.6	1.0
	Female	1531903	−30	19.0	1.0
	Male	1531502	−30	21.0	2.5
	Male	1531801	−30	25.4	2.5
	Male	1531802	−30	24.2	2.5
	Male	1531901	−30	30.6	2.5
Group 2: no treatment	Female	1531503	−30	17.6	0
	Female	1531702	−30	25.2	0
	Female	1531706	−30	25.0	0
	Female	1531803	−30	19.6	0
	Female	1531804	−30	17.8	0
	Female	1532203	−30	19.0	0
	Male	1531501	−30	32.4	0
	Male	1532101	−30	30.4	0

### Randomization

Dogs were randomized by pretreatment body weight. Sixteen (16) dogs meeting the inclusion criteria were allocated to two study groups of eight dogs each on SD −31. Dogs were assigned to study groups according to a predefined randomization chart. The dogs were ranked by SD −31 body weights in descending order (highest to lowest). The animal ID was used to break ties (highest to lowest number). The first two dogs (heaviest) were assigned to block 1, the next two dogs assigned to block 2, and so forth, until the final two dogs (lowest body weights) were assigned to the final block. Within each block, the randomization chart was created such that each dog within each block had equal chance at being assigned to one of the two treatment groups.

### Masking

Randomization of dogs to study groups and administration of the 10% imidacloprid + 2.5% moxidectin topical solution was performed by an unmasked designated person at the study facility. Witnesses to these procedures were also not masked. After randomization, the designate and witnesses were not actively involved in any other experimental procedures. All other people involved in the study execution were masked to the study group allocation.

### Inoculation with heartworm larvae

The study animals were inoculated subcutaneously on SD 0 with 50 infective, third-stage (L3) *D. immitis* larvae (Missouri isolate) harvested from infected mosquitoes (*Aedes aegypti*; Liverpool strain). This susceptible heartworm isolate originated from a naturally infected dog from northwest Missouri with no known history of treatment with macrocyclic lactones (Personal communication, Dr. Byron Blagburn, Auburn University, 2017). A blood sample positive for *D. immitis* microfilariae was originally collected from the donor dog on July 13, 2010 and was used to infect mosquitoes. The isolate was first validated in April 2011 via microfilarial testing, antigen testing, and heartworm recovery and was maintained at the College of Veterinary Medicine, Auburn University at the time of this study (Personal communication, Dr. Byron Blagburn, Auburn University, 2017). The infective third-stage larvae used for this study were harvested from mosquitoes 16 days after membrane feeding on a heparinized blood sample collected from a dog at Auburn University.

### Postmortem examination

Study animals were humanely euthanized on SD 148 with Beuthanasia-D® [Schering-Plough Animal Health (now Merck Animal Heath)] administered intravenously, 148 days after infection with third-stage *D. immitis* larvae. At necropsy adult heartworms were recovered, identified to gender, and counted. For heartworm collection, the right atrium was dissected through the main pulmonary artery. Next, each branching artery was dissected down to their furthest extent in each lung lobe. Finally, the other three heart chambers were dissected and examined for recovery of heartworms.

### Statistics for efficacy determination

Descriptive statistics (number of animals positive, geometric mean and arithmetic mean heartworm counts per animal per group) were calculated for the *D. immitis* burdens of all study groups. The heartworm counts were used to evaluate the efficacy of the 10% imidacloprid + 2.5% moxidectin topical solution (the Investigational Veterinary Product, IVP) against *D. immitis*. Percent efficacy was calculated as follows:

$$ \%\mathrm{Effectiveness}\ \left(\mathrm{reduction}\right)=\left(\mathrm{N}2-\mathrm{N}1\right)/\mathrm{N}2\times 100 $$

N1 = Geometric mean count of *D. immitis* for Group 1 treated with IVP.

N2 = Geometric mean count of *D. immitis* for the nontreated Group 2.

In accordance with the VICH GL19 guidelines, the following criteria were to be met to confirm effectiveness of the IVP for heartworm treatment and prevention.

- A minimum of six infected nontreated dogs (at least five adult heartworms recovered at necropsy per dog to substantiate adequate infection). Because this requirement was not met, a minimum of six infected nontreated dogs with at least one adult heartworm at necropsy per dog was considered as adequate infection.
- Percent efficacy for the IVP group must be 100%.
- A statistically significant difference in the number of heartworms at necropsy was needed between the treated group (Group 1) as compared with the nontreated group (Group 2).

A non-parametric statistical analysis (Wilcoxon’s Rank Sum Test) was used to test for group differences in heartworm counts using a 5% significance level. SAS Statistical Software version 9.3 was used to analyze the data from this study.

## Results

### Efficacy of treatment

Adult heartworms were not recovered from any of the eight 10% imidacloprid + 2.5% moxidectin topical solution–treated dogs in Group 1. Adult heartworms were recovered, however, from six of the eight nontreated dogs in Group 2. Overall for the nontreated dogs, a total of 134 adult *D. immitis* (range = 0–33 per dog) were recovered at necropsy (Table 2); the geometric mean *D. immitis* count for this group was 8.0 heartworms and the arithmetic mean was 16.8 heartworms (Table 3). Thus the nontreated group’s infection was considered adequate for determining efficacy of treatment. Percentage efficacy was determined to be 100%. No treatment-related adverse events were observed in any dogs.

**Table 2:** Adult heartworms, *Dirofilaria immitis*, recovered 148 days after infection with third-stage larvae from eight dogs treated with 10% imidacloprid + 2.5% moxidectin (Advantage Multi®) 30 days prior to infection and from eight untreated control dogs

Treatment group	Dog	Adult *Dirofilaria immitis*		
		Males	Females	Total
Group 1: 10% imidacloprid + 2.5% moxidectin	1531603	0	0	0
	1561903	0	0	0
	1531806	0	0	0
	1531805	0	0	0
	1531901	0	0	0
	1531801	0	0	0
	1531802	0	0	0
	1531502	0	0	0
Total worms recovered from treated dogs		0	0	0
Group 2: no treatment	1531702	9	18	27
	1531706	0	0	0
	1531803	12	7	19
	1532203	0	0	0
	1531804	13	20	33
	1531503	15	13	28
	1531501	11	14	25
	1532101	2	0	2
Total worms recovered from control dogs		62	72	134
Mean worms recovered from control dogs		7.8	9.0	16.8

**Table 3:** Adult *Dirofilaria immitis* recovered – Descriptive summary by group

Treatment group	Number of dogs			Heartworms per group		
	Per group	Positive for heartworms	With ≥5 heartworms	Geometric mean	Arithmetic mean	Median
Group 1: 10% imidacloprid + 2.5% moxidectin	8	0	0	0.0	0.0	0
Group 2: no treatment	8	6	5	8.0	16.8	22

### Heartworm antigen

All blood samples collected on SD 7 and SD 120 were negative for the presence of *D. immitis* antigens, indicating no prior or unknown exposure to heartworm infection. On SD 147, one control dog, Dog #1531503, was positive on the heartworm antigen test; this dog had a total of 28 heartworms at necropsy. All dogs in both groups were negative for microfilariae at all three time points.

## Discussion

In this study, a single dose of 10% imidacloprid + 2.5% moxidectin topical solution administered 30 days prior to heartworm infection was 100% efficacious in protecting the treated dogs from infection with this Missouri isolate of *D. immitis.* The results reported here were suggested as a possibility in an earlier publication with dogs that were treated four times every 28 days and then infected with this same isolate 28 days after the last treatment [4]. Similarly, research in cats treated with four topical doses of 10% imidacloprid + 1.0% moxidectin confirmed that after steady state was reached, all cats were protected all month long against incoming susceptible heartworm infective-stage larvae administered 7, 14, 21, and 28 days after the last treatment [3].

Moxidectin is a highly lipophilic macrocyclic lactone that is distributed and stored mainly in fat tissues [6] with a gradual elimination from the host. With the repeated application of topical 10% imidacloprid + 2.5% moxidectin for 4 months, moxidectin reaches a steady state and maintains a high plasma concentration between doses (mean concentration of 20 μg/L at 35 days after the last application) in comparison to the peak concentration after a single dose (15 μg/L) [4]. Due to the unique pharmacokinetics and high moxidectin serum concentrations maintained between monthly administrations of 10% imidacloprid + 2.5% moxidectin topical solution [4], any heartworm infective-stage larvae entering the dog during the month after administration are likely to be prevented from developing or reaching the lungs to become adult worms. This is in contrast to other products with short half-lives that are rapidly eliminated from the animal, allowing for the establishment of an infection and the development of larvae between monthly doses [7, 8].

Not all heartworm preventive products are the same, especially in regards to resistant isolates, which can be attributed to the unique pharmacokinetic properties of topical moxidectin. In the work performed as part of the original approval for topical 10% imidacloprid + 2.5% moxidectin, a single treatment was 100% effective in protecting dogs against susceptible *D. immitis* strains [9]. Several other post-approval studies have evaluated the efficacy against resistant isolates with striking differences between products. For example, in one study, a single topical treatment of 10% imidacloprid + 2.5% moxidectin was 100% effective against the resistant JYD-34 isolate [9]; this was in contrast to an efficacy of 72% for milbemycin oxime (in NexGard Spectra®; Merial) even after six monthly treatments in a second study [10]. This killing ability, even against resistant isolates, is likely due to the distinctive properties of topical 10% imidacloprid + 2.5% moxidectin, which remains in the animal during the entire month between treatments so the animal is primed with drug at the time of infection. While a single topical treatment reaches plasma concentrations of 15 μg/L and has proven to be effective against multiple resistant strains [9], when an animal is maintained on monthly topical prevention with 10% imidacloprid + 2.5% moxidectin, a plasma concentration of ≥20 μg/L is maintained between monthly doses and extends for an entire month after the last application [4]. Therefore, there is every reason to suggest that dogs should be fully protected during this entire window against incoming susceptible and resistant heartworm isolates such as those shown to survive and develop following six monthly treatments with milbemycin oxime or other products that do not undergo steady-state phenomena [7–11]. If owners forget to give a monthly application, they should administer the next treatment as soon as they recognized that they have missed a dose, and then resume the originally scheduled monthly application routine as rapidly as possible to reinstitute the steady-state protection.

## Conclusion

A single topical application of 10% imidacloprid + 2.5% moxidectin topical solution (Advantage Multi® for Dogs) administered per label 30 days prior to challenge was 100% efficacious in preventing the development of third-stage larvae of the Missouri isolate of *D. immitis* in dogs. A monthly regimen of 10% imidacloprid + 2.5% moxidectin topical solution appears to provide protection against the development of heartworms throughout the entire month between applications and not just for the 30 days prior and one to several days following product administration. The American Heartworm Society has suggested that poor owner compliance in adhering to this 30-day dosing interval is the leading cause for lack of efficacy of heartworm preventives [12]. Due to the shorter half-lives and lack of any evidence that other monthly heartworm preventives develop a protective steady state, strict adherence to their 30-day dosing interval is required in order to ensure efficacy as per label instructions. Based on this study and prior research on the steady-state phenomenon that occurs after the regular monthly application of Advantage Multi® for Dogs [4], the endpoint of protection is longer than 30 days and indistinct; if a dose is missed by a few days, there is still sufficient product in the dog to be protective. Therefore, because owners sometimes inadvertently forget or cannot apply prevention on the same day every month, Advantage Multi® for Dogs should provide more peace of mind to owners and veterinarians. The monthly use of heartworm preventive products, however, such as Advantage Multi for Dogs®, should always be recommended.

## Abbreviations

IACUC: Institutional Animal Care and Use Committee
IVP: Investigational Veterinary Product
SD: Study day
